# Prevalence and Determinants of Suboptimal Vitamin D Levels in a Multiethnic Asian Population

**DOI:** 10.3390/nu9030313

**Published:** 2017-03-22

**Authors:** Ryan Eyn Kidd Man, Ling-Jun Li, Ching-Yu Cheng, Tien Yin Wong, Ecosse Lamoureux, Charumathi Sabanayagam

**Affiliations:** 1 Singapore Eye Research Institute, the Academia, 20 College Road, Discovery Tower Level 6, Singapore 169856, Singapore; man.eyn.kidd.ryan@seri.com.sg (R.E.K.M.); queenie.li.l.j@seri.com.sg (L.-J.L.); cheng.ching.yu@seri.com.sg (C.-Y.C.); wong.tien.yin@singhealth.com.sg (T.Y.W.); ecosse.lamoureux@seri.com.sg (E.L.); 2 Ophthalmology and Visual Sciences Academic Clinical Program, Duke-NUS Medical School, Singapore 169857, Singapore; 3 Department of Ophthalmology, National University of Singapore, Singapore 119228, Singapore; 4 Centre for Quantitative Medicine, Duke-NUS Medical School, Singapore 169856, Singapore

**Keywords:** ageing population, ethnicity, vitamin D, vitamin D deficiency, vitamin D insufficiency

## Abstract

This population-based cross-sectional study examined the prevalence and risk factors of suboptimal vitamin D levels (assessed using circulating 25-hydroxycholecalciferol (25(OH)D)) in a multi-ethnic sample of Asian adults. Plasma 25(OH)D concentration of 1139 Chinese, Malay and Indians (40–80 years) were stratified into normal (≥30 ng/mL), and suboptimal (including insufficiency and deficiency, <30 ng/mL) based on the 2011 Endocrine Society Clinical Practice Guidelines. Logistic regression models were used to assess the associations of demographic, lifestyle and clinical risk factors with the outcome. Of the 1139 participants, 25(OH)D concentration was suboptimal in 76.1%. In multivariable models, age ≤65 years (compared to age >65 years), Malay and Indian ethnicities (compared to Chinese ethnicity), and higher body mass index, HbA1c, education and income levels were associated with suboptimal 25(OH)D concentration (*p* < 0.05). In a population-based sample of Asian adults, approximately 75% had suboptimal 25(OH)D concentration. Targeted interventions and stricter reinforcements of existing guidelines for vitamin D supplementation are needed for groups at risk of vitamin D insufficiency/deficiency.

## 1. Introduction

Vitamin D is critical to bone and mineral metabolism [1], as well as extra-skeletal processes such as cell proliferation and apoptosis, regulation of renin production [2], and immunomodulation [3]. Vitamin D, from either cutaneous synthesis or dietary intake, undergoes hydroxylation in the liver into 25-hydroxy vitamin D (25(OH)D), the most abundant circulating form of vitamin D [4]. Circulating 25(OH)D is widely regarded as reflecting the sum of cutaneous synthesis and oral intake, and has been recognised as being the best functional indicator of vitamin D status [4].

Previous research has linked vitamin D deficiency, defined as 25(OH)D concentration ≤20 ng/mL , to increased risk of rickets, osteomalacia and osteoporotic bone fractures [1,5,6]. There have also been data showing vitamin D deficiency to be associated with cardiovascular disease [7], cancer [7], diabetes [8], cognitive impairment [9], and all-cause mortality [10], although evidence for these non-skeletal conditions remain inconclusive [11]. These detrimental effects are not limited to persons with deficient vitamin D levels, as a systematic review found that merely having an insufficient 25(OH)D concentration (21–29 ng/mL [12]) predisposed women to breast cancer, and poorer survival rates in those diagnosed with lymphoma [13].

In Asia, there is growing concern regarding the potential for vitamin D insufficiency and deficiency. The main sources of vitamin D in Asia include fish, meat, fortified dairy products, eggs, liver and sundried mushrooms. However, there have recently been concerted efforts by public health programs to advocate limiting the intake of foods high in saturated fats that happen to be rich sources of vitamin D (e.g., eggs and offal). In addition, dietary diversity tends to be narrow particularly during a typical workday [10] due to the limited amount of time for meal preparation and consumption, which may exacerbate the issue. Cutaneous production of vitamin D also decreases with age [14], and Asia has some of the fastest aging populations in the world [15]. Moreover, vitamin D synthesis has been shown to be inhibited in persons with darker pigmentation [16], due to absorption and scattering of ultraviolet light by melanin [17]; and pertinent to this, a substantial proportion of Asians have dark skin, in particular those of Malay and Indian ethnicities. Additionally, the rapid economic development in the past few decades has resulted in young and middle-aged adults mainly having indoor jobs [18], hence limiting exposure to sunlight and consequently decreasing cutaneous synthesis of vitamin D.

To date, although various studies have assessed the prevalence and determinants of vitamin D insufficiency and deficiency, the data reported are applicable to only a single ethnic group, e.g., Indians [19], and Chinese [20]. The impact of ethnicity and ingrained culture on vitamin D levels across a multi-ethnic population, where all individuals have the same access to healthcare and services hence enabling direct comparisons, are quite limited. This is especially important in Southeast Asia, with its rich ethnic and cultural diversity. For instance, in Islam, the traditional attire for females emphasizes modesty, exposing only the face and hands; it is therefore unsurprising that lower 25(OH)D concentration was found in females compared to males in Malaysia, where the main religion is Islam [21]. In terms of the effect of ethnicity on 25(OH)D concentration, only clinical studies performed on specific target populations (e.g., hospitalized elderly patients [22]) are available; as such, information on vitamin D status and determinants of insufficiency/deficiency in populations at risk (e.g., community-dwelling middle-aged and older adults) of developing vitamin D deficiency-related complications such as osteoporosis and cardiovascular disease in Asia are limited. 

While guidelines on vitamin D intake and supplementation in Asian populations may be quite comprehensive, e.g., Singapore’s dietary guidelines [23] which recommends vitamin D supplementation if an individual wears clothing that covers the entire body, is of south Indian descent, or stays mostly indoors, these are usually not reinforced given the reticence of elderly Asians to visit their doctors due to language barriers or for fear of hearing “bad news” with regards to their health [24]. In this study, we therefore investigated the prevalence and determinants of suboptimal 25(OH)D concentration in a multi-ethnic sample of adults in Singapore.

## 2. Subjects and Methods

### 2.1. Study Population

Data for the present study were derived from controls from a previous case-control study (Singapore Kidney-Eye Study, *n* = 2944) nested within the Singapore Epidemiology of Eye Diseases (SEED) study, a large epidemiological study comprising of data from the three major ethnic groups in Singapore: Malays (Singapore Malay Eye Study, 2004–2006), Indians (Singapore Indian Eye Study, 2007–2009), and Chinese (Singapore Chinese Eye Study, 2009–2011). All studies followed the same study protocol, were conducted in the same study centre (Singapore Eye Research Institute), and recruited adults aged 40–80 years residing in the southwestern part of Singapore though an age-stratified random sampling method. The SEED methodology and population characteristics have been published elsewhere [25]. Subjects participating in the study were included after having given their written informed consent for research. The study was conducted in accordance with declaration of Helsinki and both the SEED study and Singapore Kidney Eye Study were approved by the Singapore Eye Research Institute Institutional Review Board. All participants underwent a clinical, questionnaire-based, and laboratory examination at the study centre.

In the Singapore Kidney-Eye Study, cases were those with chronic kidney disease (CKD) or age-related macular degeneration (AMD). Controls were those without CKD or AMD, age, sex and ethnicity matched to cases. For the current analysis, we excluded the cases (*n* = 1628) and included only the control participants (*n* = 1316). Except for the absence of CKD and AMD, controls were not restricted by other comorbid or chronic conditions such as diabetes, hypertension, asthma, etc. After further excluding those with missing data on important covariates including blood pressure, blood glucose and plasma lipids (*n* = 177), 1139 participants were included for the final analysis.

### 2.2. Assessment of Circulating 25(OH)D Concentration

Circulating 25(OH)D level was measured from venous blood collected in the non-fasting state at baseline and stored at −80 °C. The collection and storage process was the same for all study participants. Serum samples were used in Chinese and Indians and plasma samples were used in Malays for measuring 25(OH)D. Circulating 25(OH)D concentration was measured using electrochemiluminescence immunoassay [26] at the National University Hospital Reference laboratory accredited by the College of American Pathologists, which is part of the VDSP (Vitamin D Standardization Program). Quality control checks were performed using materials provided by ROCHE Diagnostics. The lower limit of detection of 25(OH)D in the current study was 4 ng/mL and the coefficient of variation of the assay ranged between 4.7%–11.5% for a concentration range between 10.3 and 57.5 ng/mL. All samples were processed continuously in two batches over a period of 3 months. 25(OH)D concentration was categorized as follows: optimal (≥30 ng/mL), insufficient (21–29 ng/mL), and deficient (<20 ng/mL), as defined by the 2011 clinical practice guidelines recommended by the Endocrine Society [12].

### 2.3. Assessment of Covariates

An interviewer-administered standardized questionnaire was used to obtain information on socio-demographic and lifestyle factors including age, gender, ethnicity, history of regular smoking, alcohol consumption, highest education level attained, income, and nature of occupation [25,27]. High school education was defined as ≥6 education years. Regular alcohol consumption was defined as ≥5 days a week. Outdoor occupations (as a proxy for outdoor sunlight exposure) included agricultural worker, production craftsman, transportation driver, shipping and maritime worker, postal and dispatch delivery worker, and labourer/odd job labourer. Anthropometric and blood pressure measurements were obtained from a standardized physical examination [25,27]. Height was measured in centimetres using a wall-mounted measuring tape and weight in kilograms using a digital scale after participants were instructed to remove shoes and heavy objects such as belts, phones, keys, and wallets. Body mass index (BMI) was calculated as the weight in kilograms divided by the square of height in meters (kg/m^2^) and categorized into underweight (<18.5 kg/m^2^), normal (18.5–24.9 kg/m^2^), overweight (25–29.9 kg/m^2^), and obese (≥30 kg/m^2^) according to WHO-defined BMI cut points [28]; however, due to the small sample size of individuals who were underweight (*n* = 62), we combined the underweight and normal weight categories. Systolic blood pressure (SBP) and diastolic blood pressure (DBP) were measured using a digital automatic blood pressure monitor after the participant was seated for at least 5 minutes and an average of two measurements were taken as the blood pressure value for that individual. Circulating HbA1c levels were determined using automated standard laboratory methods at the National University Hospital laboratory. Diabetes mellitus was identified from plasma glucose of 200 mg/dL (11.1 mmol/L) or more, self-reported use of diabetic medication, or physician-diagnosed diabetes. Hypertension was considered present if systolic and diastolic blood pressure were ≥140/90 mmHg, there was self-reported use of anti-hypertensive medication, or if physician-diagnosed hypertension was present. Finally, hyperlipidaemia was identified from total cholesterol >6.2 mmol/L, self-reported use of lipid-lowering medication, or physician-diagnosed hyperlipidaemia.

### 2.4. Statistical Analyses

All analyses were performed using Intercooled Stata version 12.1 for Windows (StataCorp, Lake Station, TX, USA). Prevalence was calculated as the number of persons with suboptimal 25(OH)D concentration (i.e., insufficiency and deficiency) divided by total number of participants. Prevalence was also calculated stratified by gender and ethnic groups. We then investigated whether there were any significant associations of specific characteristics of participants with normal and suboptimal concentration of 25(OH)D using the Chi-square statistic for categorical, and independent sample *t*-test for continuous variables. Risk factors examined were chosen based on previous demonstrated relationships with 25(OH)D concentration from published literature. We then examined associations of risk factors (exposures) with presence of suboptimal 25(OH)D concentration (outcome) using two logistic regression models: (1) adjusted for age and gender; and (2) additionally adjusted for ethnicity, socioeconomic (education, income and having an outdoor job), lifestyle (BMI, smoking status, and alcohol consumption) and clinical variables (presence of diabetes, hypertension and hyperlipidaemia status; systolic blood pressure; and HbA1c levels). We examined statistical interaction between all biological categorical variables (age, gender, ethnicity and BMI) by including cross-product interaction terms in the corresponding regression model. In addition, we conducted sub-group analyses to evaluate the determinants of 25(OH)D insufficiency and deficiency separately by utilizing multinomial logistic regression models. Significance was defined as *p* < 0.05 (two-tailed). As Singapore lies close to the equator with minimal seasonal variations, no seasonal stratification of data was conducted.

## 3. Results

Of the 1139 participants 45.2% female; the mean (standard deviation, SD) age was 67 (9) years (age range 41–80 years) and mean (SD) 25(OH)D concentration was 22.1 (10.5) ng/mL. Prevalence of suboptimal 25(OH)D concentration in the overall population was 76.1% (73.6%–78.5%), comprising 28.4% with insufficiency and 47.8% with deficiency. Women had higher prevalence of suboptimal 25(OH)D concentration compared to men (88.7% vs. 65.7%, *p* < 0.001). Among the three ethnic groups, Indians (84.3%) and Malays (84.1%) had significantly higher prevalence of suboptimal 25(OH)D concentration compared to Chinese (52.3%; *p* < 0.001). Table 1 shows the characteristics of the participants by 25(OH)D status. Participants with suboptimal 25(OH)D concentration were more likely to be younger, female, Malays or Indians, had higher BMI and HbA1c levels; less likely to be current or ex-smokers, alcohol drinkers, and had higher prevalence of diabetes as compared to persons with normal 25(OH)D concentration.

**Figure 1:**
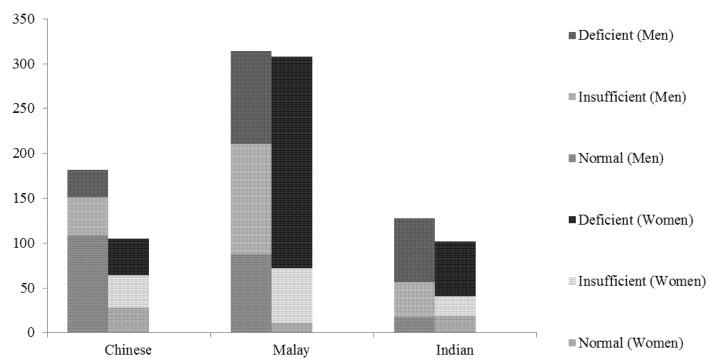
Bar graph showing 25(OH)D status by gender and ethnicity.

In Chinese and Malay ethnicities, the prevalence of suboptimal 25(OH)D concentration was significantly higher in women compared to men, In Indians, prevalence of suboptimal 25(OH)D concentration was similar between men and women (Figure 1).

In the age and gender adjusted model (Table 2, Model 1), age ≤ 65 years, female gender, Malay and Indian ethnicities, higher levels of BMI, HbA1c levels, and systolic BP, and presence of diabetes were significantly associated with suboptimal 25(OH)D concentration. In contrast, regular alcohol consumption was associated with lower odds of having suboptimal 25(OH)D. In multivariable adjusted models, the above associations were largely unchanged, although systolic BP and presence of diabetes became attenuated while higher education (≥high school) and income levels (>SGD$2000) became significantly associated with increased odds of having suboptimal 25(OH)D concentration (Table 2, Model 2).

In analyses including interaction terms between variables (age, gender, ethnicity and BMI) for suboptimal 25(OH)D concentration, all were non-significant (*p* > 0.1) except for an interaction between gender and ethnicity (*p* < 0.001). We therefore conducted ethnicity-stratified and multivariable adjusted analyses of the association between gender and suboptimal 25(OH)D concentration and found that the association between female and suboptimal 25(OH)D concentration was significant in Chinese and Malay ethnicities, but not in Indians (Table 3).

In subgroup analyses stratified by 25(OH)D insufficiency and deficiency, female gender, Malay and Indian ethnicities and higher income were associated with greater odds of 25(OH)D insufficiency, while regular alcohol consumption was inversely associated with 25(OH)D insufficiency (Table 3). Age ≤65 years, female gender, higher BMI levels, Malay and Indian ethnicities, higher education levels, and greater HbA1c levels were associated with 25(OH)D deficiency. Regular alcohol consumption, similar to insufficiency outcome, was inversely associated with deficiency as well (Table 4).

## 4. Discussion

In this study, we found that approximately a quarter of Asian adults had 25(OH)D insufficiency and almost half were 25(OH)D deficient. We further demonstrated that age ≤65 years, female gender, Malay or Indian ethnicity, and having higher BMI, HbA1c, education and income levels, were associated with suboptimal 25(OH)D concentration. If confirmed in future prospective studies, our results suggest that stricter reinforcement of public health guidelines on vitamin D supplementation for at-risk groups may be needed for Asian adults ≥40 years of age.

While studies have been done on the 25(OH)D status of Chinese and Indian ethnicities in isolation (i.e., China and India, respectively), it is difficult to compare the prevalence and risk factors due to the differences in 25(OH)D classification systems utilized across the different studies [19,20]. For instance, Babu and colleagues utilized a cut-off of 50 nmol/L for insufficiency (~20 ng/mL) while Lu and associates instead defined insufficiency as 75 nmol/L (~30 ng/mL). Our results, using the current recommended guidelines of <30 ng/mL to define suboptimal 25(OH)D concentration [12], revealed an inverse relationship of vitamin D levels with age. Due to Singapore’s ageing population [29] and a corresponding decline in cutaneous vitamin D production [14], the inverse would have been true, i.e., we would expect a linear decline in 25(OH)D concentration with age. There may be several reasons underlying this result, much of which can be attributed to the fact that approximately half of Singaporean workers hold indoor jobs, which may have limited their exposure to sunlight [29]. Stratification of our sample population into working age adults (≤65 years) and retired adults (>65 years) reveal that working-aged adults were more likely to be 25(OH)D deficient, hence supporting the above hypothesis. However, older adults may also be more likely to be taking supplements (e.g., multivitamins) containing vitamin D. As we did not collect supplement or dietary data in our sample, further studies may be warranted to elucidate the effects of diet and supplement use on the inverse age-25(OH)D relationship.

We also observed that women were more than twice as likely to be 25(OH)D insufficient, and almost five times more likely to be 25(OH)D deficient compared to men. Further analyses revealed however, that this gender discrepancy was evident only in Chinese and Malay ethnicities. This could be due to various factors, chief among which is the fact that Asian Chinese women tend to subscribe to the notion that “fairer is beautiful” and tend to avoid exposure to the sun as a result [30]. Moreover, the main religion of Malays is Islam, which as mentioned previously, emphasizes modesty, particularly in women. The traditional female Muslim attire, comprising a headscarf and long dresses that leave only the face and hands exposed, may hence block ambient sunlight, leading to a decrease in cutaneous vitamin D production and contributing towards lower 25(OH)D concentration compared to men. In addition, it may be plausible that since women have higher body fat percentages compared to men, the bioavailability of vitamin D, a fat-soluble vitamin, may be reduced due to sequestration in fatty tissue [4], hence contributing to the observed gender discrepancy in 25(OH)D concentration. However, our gender-stratified models have accounted for BMI as a confounder, indicating that the gender-specific differences in 25(OH)D concentration were likely to be independent of BMI. Future studies including body composition measures may help clarify this association. 

Our results also show that individuals of Malay and Indian ethnicity were associated with 25(OH)D insufficiency and deficiency as compared to individuals of Chinese ethnicity. This is not surprising given that increased skin pigmentation, specifically melanin, has been shown to scatter and absorb ultraviolet radiation [17], hence resulting in decreased cutaneous production. However, what is alarming are the extremely high odds of being vitamin D deficient if an individual is of these two ethnicities - Malays had a 6 fold increased odds of 25(OH)D deficiency, while those of Indian ethnicity had an almost 9-fold odds of having 25(OH)D deficiency. 

Having an income >SGD$2000 per month and high school or higher education (compared to primary school education or less) were associated with suboptimal 25(OH)D concentration. In comparison, previous research has shown an association between low socioeconomic status and reduced vitamin D intake [31]. We speculate that individuals in the higher socioeconomic strata are more likely to be in white-collar professions (e.g., office workers, lawyers) that largely conduct business indoors, hence limiting sunlight exposure. However, these individuals may also be more likely to take vitamin D supplements [32]. These findings must therefore be taken with caution as we did not collect dietary or supplement information, hence necessitating future cohort studies to validate our findings. 

In the current study, no significant association was found between outdoor work and suboptimal 25(OH)D concentration. This may be due to the small number of individuals with outdoor jobs (*n* = 51 (4.4%)), since more direct sun exposure is expected to increased cutaneous synthesis of vitamin D and reduce odds of 25(OH)D insufficiency and deficiency [33]. Our data also suggest that regular consumption of alcohol (≥5 days a week) was associated with increased 25(OH)D concentration. Although chronic alcoholism is known to disturb vitamin D metabolism, resulting in a low concentration of 25(OH)D [34,35], experimental animal models [36] and a recent population-based study [37] both appear to support the protective effect of regular alcohol consumption on 25(OH)D concentration. However, caution must be taken when interpreting our results as we used only a very crude measure of alcohol intake, i.e., number of times/week, and we did not assess volume (in terms of standard drinks) imbibed per occasion. Future prospective studies including detailed assessment of alcohol consumption may help clarify our findings. 

We found that obesity was independently associated with both 25(OH)D insufficiency and deficiency, which is in agreement with the results from previous research [4]. However, the exact temporal nature of this relationship is still unclear. It has been hypothesized that in obese persons, the excess fat stores might reduce the bioavailability of fat-soluble vitamin D due to sequestration in fatty tissue [4], and the increased mass and surface area in these individuals might also lower vitamin D levels via volumatric dilutation [38]. In addition, obese individuals are likely to spend more time indoors [39], hence minimizing exposure to sunlight and reducing cutaneous production of vitamin D. On the other hand, a reduction in vitamin D may lead to lowered leptin levels [40], a hormone responsible for suppressing appetite and increasing metabolism, hence resulting in unintentional weight gain and obesity. Therefore, longitudinal studies to determine the cause-effect nature and mechanisms underlying the relationship between obesity and suboptimal 25(OH)D concentration may be warranted.

Due to the close vitamin D-obesity relationship, it is believed that vitamin D deficiency contributes to the pathogenesis of diabetes and the metabolic syndrome via obesity-induced insulin resistance [41]. The results of our study support this hypothesis, as the significant association of diabetes with suboptimal 25(OH)D concentration in age-gender adjusted models became attenuated after additional adjustment for BMI and other lifestyle factors. In addition, an association between increased HbA1c and suboptimal 25(OH)D concentration independent of diabetes presence was also observed. This is expected given that vitamin D has been established to be involved in the process of insulin secretion from pancreatic beta cells [42], and decreased levels of vitamin D intake may therefore result in impaired insulin secretion, and consequently higher HbA1c levels.

Strengths of this study include the population-based nature of the data, as well as the systematic and clearly defined clinical tests utilized in the the study protocol. Some limitations are also noted. First, the cross-sectional nature of these observations do not permit us to determine the temporal sequence of events. Second, the measurement of 25(OH)D concentration was based on a one-time measure and hence, would not show any potential differences in 25(OH)D concentration at different times of the year. However, as Singapore is situated at the equatorial belt and does not experience changes in season per se, we anticipate that the differences in 25(OH)D concentration at different time periods to be minimal. Third, we did not have data on potential confounding factors including sunscreen application, clothing coverage, dietary information and vitamin D supplementation, and hence were unable to determine the impact of these factors on 25(OH)D status in our study. Finally, we did not record skin tone, which would have corroborated our hypothesis that the melanin in the skin slows down cutaneous production of vitamin D by absorbing most of the available UV light required for vitamin D synthesis. 

## 5. Conclusions

Suboptimal concentration of 25(OH)D including insufficiency and deficiency were associated with age < 65 years, female gender, Malay and Indian ethnicities, higher education and income levels, obesity, and higher HbA1c. If confirmed in future prospective studies, our results may have implications for adding to and reinforcing public health guidelines recommending safe doses of vitamin D supplementation (2000 IU or 10 μg) [43] for the populations at risk.

## Figures and Tables

**Table 1:** Clinical and demographic characteristics of sample participants by plasma 25(OH)D concentration (*n* = 1139).

Clinical and Demographic Variables	Normal (≥30 µg/L; *n* = 272)	Suboptimal (≤29 µg/L; *n* = 867)	*p*-Value *
	Mean (Standard Deviation)		
Age (years)	68.5 (8.2)	66.2 (8.7)	0.002
Systolic BP (mmHg)	144.7 (21.1)	148.2 (22.4)	0.02
Diastolic BP (mmHg)	77.9 (9.9)	78.1 (10.3)	0.7
BMI (kg/m^2^)	23.3 (3.7)	25.6 (4.9)	<0.001
HbA1c (%)	6.1 (0.9)	6.5 (1.4)	0.001
	N (%)		
Age (*n* (%))			
≤65	77 (28.3)	346 (39.9)	0.001
>65	195 (71.7)	521 (60.1)	
Gender (*n* (%))			
Male	214 (78.7)	410 (47.3)	<0.001
Female	58 (21.3)	457 (52.7)	
BMI (*n* (%))			
<25	184 (67.7)	415 (47.9)	<0.001
≥25–29.9	78 (28.7)	310 (35.8)	
≥30	10 (3.7)	142 (16.4)	
Ethnicity (*n* (%))			
Chinese	137 (50.4)	150 (17.3)	<0.001
Malay	99 (36.4)	523 (60.3)	
Indian	36 (13.2)	194 (22.4)	
Smoking (*n* (%))			
Never	141 (51.8)	589 (67.9)	<0.001
Current	65 (23.9)	119 (13.7)	
Ex	66 (24.3)	159 (18.3)	
Alcohol consumption (*n* (%))			
No	242 (89.0)	837 (96.5)	<0.001
Yes	30 (11.0)	30 (3.5)	
Education (*n* (%))			
No high school education	206 (75.7)	657 (75.8)	0.9
High school education and above	66 (24.3)	210 (24.2)	
Income (*n* (%))			
<SGD 2000	247 (90.8)	776 (89.5)	0.5
≥SGD2000	25 (9.2)	91 (10.5)	
Outdoor Job (*n* (%))			
No	256 (94.1)	832 (96.0)	0.2
Yes	16 (5.9)	25 (4.0)	
Diabetes (*n* (%))			
No	221 (81.3)	634 (73.1)	0.007
Yes	51 (18.7)	233 (26.9)	
Hypertension (*n* (%))			
No	76 (27.9)	204 (23.5)	0.1
Yes	196 (72.1)	663 (76.5)	
Hyperlipidaemia (*n* (%))			
No	143 (52.6)	446 (51.4)	0.7
Yes	129 (47.4)	421 (48.6)	

* Chi-square (categorical)/independent sample *t*-test (continuous).

**Table 2:** Associations Of clinical and demographic variables with suboptimal 25(OH)D concentration.

Parameters	Model 1 *		Model 2 ^†^	
	OR (95% CI)	*p* Value	OR (95% CI)	*p* Value ^‡^
Age (per year increase)	0.97 (0.95, 0.99)	<0.001	0.98 (0.96, 1.00)	0.055
>65	Reference	-	Reference	-
≤65	1.66 (1.22, 2.26)	0.001	1.69 (1.14, 2.52)	0.009
Gender				
Male	Reference	-	Reference	-
Female	4.13 (2.99, 5.69)	<0.001	3.92 (2.54, 6.06)	<0.001
BMI (per kg/m^2^ increase)	1.10 (1.06, 1.14)	<0.001	1.07 (1.02, 1.11)	0.002
<25	Reference	-	Reference	-
≥25–29.9	1.58 (1.16, 2.17)	0.004	1.31 (0.93, 1.86)	0.1
≥30	4.16 (2.1, 8.21)	<0.001	2.82 (1.38, 5.78)	0.005
Ethnicity				
Chinese	Reference	-	Reference	-
Malay	4.64 (3.33, 6.47)	<0.001	4.71 (3.22, 6.89)	<0.001
Indian	5.19 (3.33, 8.08)	<0.001	5.93 (3.61, 9.72)	<0.001
Smoking				
Never	Reference	-	Reference	-
Current	0.91 (0.61, 1.37)	0.7	0.84 (0.53, 1.33)	0.5
Ex	1.38 (0.93, 2.03)	0.1	1.12 (0.73, 1.73)	0.6
Alcohol Consumption, yes	0.41 (0.24, 0.71)	0.001	0.38 (0.2, 0.73)	0.004
Education, ≥high school	1.17 (0.83, 1.66)	0.4	1.57 (1.05, 2.35)	0.027
Income, >$2000	1.37 (0.83, 2.28)	0.2	2.10 (1.17, 3.76)	0.013
Outdoor Job, yes	0.87 (0.46, 1.64)	0.7	0.83 (0.41, 1.69)	0.6
Diabetes, yes	1.65 (1.16, 2.36)	0.006	0.71 (0.42, 1.21)	0.2
Hypertension, yes	1.39 (0.99, 1.94)	0.06	1.14 (0.72, 1.81)	0.6
Hyperlipidaemia, yes	0.98 (0.74, 1.31)	0.9	0.95 (0.69, 1.32)	0.8
Systolic BP, per mmHg increase	1.01 (1.00, 1.01)	0.027	1.00 (0.99, 1.01)	0.9
Diastolic BP, per mmHg increase	1.01 (0.99, 1.02)	0.3	1.00 (0.98, 1.02)	0.8
HbA1c, per % increase	1.33 (1.15, 1.55)	<0.001	1.28 (1.05, 1.55)	0.014

* Age and gender adjusted; ^†^ Includes all variables; ^‡^ Logistic regression models.

**Table 3:** Multivariable adjusted * associations of gender with suboptimal 25(OH)D concentration stratified by ethnicity.

	Chinese	Malay	Indian
Gender	OR (95% CI)	OR (95% CI)	OR (95% CI)
Male	Reference	Reference	Reference
Female	4.74 (2.34, 9.60)	9.11 (3.99, 20.7)	0.46 (0.16, 1.36)

* Adjusted for age, gender, BMI, smoking, alcohol consumption, education, monthly income, outdoor job, presence of diabetes, hypertension and hyperlipidaemia, systolic and diastolic blood pressure, HBA1c.

**Table 4:** Multivariable adjusted associations of clinical, demographic and lifestyle parameters with 25(OH)D insufficiency and deficiency.

Parameters	Insufficiency (21–29 ng/mL)		Deficiency (≤20 ng/mL)	
	OR (95% CI)	*p* Value	OR (95% CI)	*p* Value *
Age (per year increase)	0.99 (0.96, 1.01)	0.4	0.97 (0.95, 0.99)	**0.012**
>65	Reference		Reference	
≤65	1.34 (0.86, 2.08)	0.2	2.04 (1.33, 3.14)	0.001
Gender				
Male	Reference		Reference	
Female	2.49 (1.53, 4.06)	<0.001	5.47 (3.40, 8.81)	<0.001
BMI (per kg/m^2^ increase)	1.04 (1.00, 1.09)	0.07	1.09 (1.04, 1.14)	<0.001
<25	Reference		Reference	
≥25–29.9 (overweight)	1.19 (0.81, 1.75)	0.4	1.43 (0.97, 2.09)	0.07
≥30 (obesity)	1.76 (0.80, 3.86)	0.2	3.73 (1.78, 7.84)	0.001
Ethnicity				
Chinese	Reference		Reference	
Malay	3.40 (2.22, 5.21)	<0.001	6.47 (4.17, 10.05)	<0.001
Indian	3.81 (2.19, 6.62)	<0.001	8.72 (5.04, 15.11)	<0.001
Smoking				
Never	Reference		Reference	
Current	0.93 (0.56, 1.56)	0.8	0.77 (0.46, 1.3)	0.3
Ex	1.38 (0.86, 2.23)	0.2	0.9 (0.55, 1.47)	0.7
Alcohol Consumption, yes	0.30 (0.14, 0.67)	0.003	0.47 (0.22, 0.99)	0.047
Education, ≥high school	1.43 (0.91, 2.24)	0.1	1.75 (1.12, 2.73)	0.013
Income, >$2000	2.39 (1.27, 4.5)	0.007	1.83 (0.96, 3.48)	0.065
Outdoor Job, yes	1.21 (0.57, 2.56)	0.6	0.53 (0.23, 1.25)	0.1
Diabetes, yes	0.74 (0.41, 1.33)	0.3	0.7 (0.4, 1.24)	0.2
Hypertension, yes	1.29 (0.77, 2.16)	0.3	1.03 (0.62, 1.72)	0.9
Hyperlipidaemia, yes	0.92 (0.64, 1.31)	0.6	0.99 (0.7, 1.42)	0.9
Systolic BP (per mmHg increase)	1.00 (0.99, 1.01)	0.9	1.00 (0.99, 1.01)	0.9
Diastolic BP (per mmHg increase)	0.99 (0.97, 1.02)	0.6	1.00 (0.98, 1.02)	0.9
HbA1c (per % increase)	1.21 (0.98, 1.49)	0.08	1.33 (1.09, 1.64)	0.006

* Multinomial logistic regression models.
